# Prediction of Relapse and Steroid Dependency in Pediatric Ulcerative Colitis

**DOI:** 10.3390/medicina62010045

**Published:** 2025-12-25

**Authors:** Mehmet Onder, Cigdem Omur Ecevit, Safak Pelek, Duygu Demirtas Guner, Gulin Eren, Sevim Cakar, Ozlem Bekem

**Affiliations:** 1 Department of Pediatric Gastroenterology, Hepatology and Nutrition, University of Health Sciences Dr. Behcet Uz Children’s Hospital, Izmir 35210, Türkiye; ctecevit@hotmail.com (C.O.E.); safakpelek@hotmail.com (S.P.); duygudemirtas@gmail.com (D.D.G.); gulinerdemir@yahoo.com (G.E.); obekem@yahoo.com (O.B.); 2 Department of Pediatric Gastroenterology, Hepatology and Nutrition, Faculty of Medicine, Dokuz Eylul University, Izmir 35330, Türkiye; drsevimgokgoz@gmail.com

**Keywords:** ulcerative colitis, IgM, MMES, prediction, steroid dependence

## Abstract

*Background and Objectives*: The objective of this study is to ascertain the predictive criteria for steroid dependence and relapse in patients diagnosed with ulcerative colitis. Additionally, the study aims to provide data that will enable earlier transition to second-line treatment when necessary. *Materials and Methods*: The study included 62 patients diagnosed with ulcerative colitis between 2018 and 2023, who were followed up at the Department of Pediatric Gastroenterology, Hepatology, and Nutrition at the University of Health Sciences, Izmir Dr. Behçet Uz Children’s Hospital. Demographic data included age and gender at diagnosis, BMI, weight-for-age, and height-for-age. Laboratory parameters recorded were complete blood count, total protein, albumin, CRP, ESR, IgG, IgM, IgA, IgG subclasses, vitamin D, B12, folic acid, and ferritin levels. *Results*: The study included 62 patients. Thirty-two patients (51.6%) were female. In the univariate regression analysis, there was an inverse correlation between IgM levels and relapse and steroid dependence (*p* < 0.01, *p* = 0.03, respectively). Additionally, a relationship was identified between steroid dependence and hemoglobin, hematocrit, white blood cell count, neutrophil count, platelet count, and albumin levels (*p =* 0.01, *p* < 0.01, *p* < 0.01, *p =* 0.01, *p* < 0.01, *p =* 0.03, respectively). There was a significant relationship between MMES and steroid dependence (*p* < 0.01). MMES was found to be significant in predicting steroid dependence in patients with pancolitis (AUC: 0.75, 95% CI: [0.60–0.90], *p =* 0.01). *Conclusions*: We conclude, as for Crohn’s disease, an algorithm or a specific scoring system for ulcerative colitis is needed for the use of anti-TNF drugs as first-line treatment in pediatric ulcerative colitis. The initial severity of the disease appears to be the most important risk factor in terms of steroid dependence. Based on our study and the literature data, a scoring system incorporating parameters such as hemoglobin, hematocrit, WBC, albumin, platelet, and IgM levels, disease involvement type, initial PUCAI score, and MMES would be prudent to adopt.

## 1. Introduction

Ulcerative colitis is a chronic inflammatory disease that begins in the rectum and progresses proximally without interruption, and is characterized by relapses [1]. The prevalence of the disease varies by country, ranging from 1 to 20 per 100,000, and the incidence of the disease is increasing annually [2,3].

Today, we still do not fully understand the pathophysiology of the disease. One observation is that there is a breakdown of the mucosal barrier which activates neutrophils, macrophages and natural killer cells. An additional observation is the triggering of the adaptive immune response [4,5]. Genetic, microbiota and environmental factors also play a role in the onset of the disease, along with this immune response [4].

The most common symptoms of the disease are often bloody diarrhea, chronic abdominal pain, weight loss, and developmental delay. It may also present with arthritis, uveitis, erythema nodosum, and primary sclerosing cholangitis [6,7].

The diagnosis of ulcerative colitis is made through a comprehensive evaluation of clinical findings, laboratory markers, endoscopy results, and histopathological examination findings. The updated Porto criteria, comprising 23 evaluation criteria including endoscopy data, both macroscopic and histopathological, clinical findings, and laboratory parameters, serve as a guide for the diagnosis of ulcerative colitis [8]. To define the spread of the disease, it was recommended that the Montreal Classification initially be used, but the revised Paris Classification is now the recommendation [9,10].

The PUCAI score is another method used to determine the severity of the disease at diagnosis. It evaluates the clinical findings for the purpose of classifying patients into four groups: remission, mild, moderate, and severe [11,12,13]. ESPGHAN’s treatment algorithm is based on the PUCAI score of the patient at diagnosis. For mild to moderate disease without systemic findings, the algorithm recommends high-dose oral 5-ASA and enemas for proctitis. For severe colitis, the algorithm recommends oral or intravenous steroid therapy in addition to systemic findings [12,14].

The use of anti-TNF drugs, such as infliximab, adalimumab, and golimumab, is recommended in patients who do not respond to steroid treatment, who experience an attack while continuing steroid treatment, or who experience an attack shortly after discontinuing steroid treatment. If these treatments are ineffective, recommended therapies would turn to anti-integrin (vedolizumab) and anti-IL23 (ustekinumab) [14]. Dual biological agent therapies may also be administered to prevent the need for surgical interventions [15]. If medical treatments are ineffective, surgical treatment should be considered [14].

During an inflammatory bowel disease flare-up, the primary objective is to promptly suppress disease activity. In the case of Crohn’s disease, when the diagnosis is made in a high-risk situation—that is, in the presence of severe growth retardation, perianal disease, stricture, or widespread and deep ulcers—it is recommended that treatment with anti-TNF drugs be initiated without delay, prior to the administration of first-line treatments [16]. Previous studies have demonstrated associations between disease severity and factors such as anemia at diagnosis, hypoalbuminemia, and higher PUCAI scores; however, a clear and definitive recommendation for ulcerative colitis cannot yet be established [17,18,19,20,21,22]. The objective of this study is to ascertain the predictive criteria for steroid dependence and relapse in patients diagnosed with ulcerative colitis. Additionally, the study aims to provide data that will enable earlier transition to second-line treatment when necessary.

## 2. Materials and Methods

The study included 118 patients diagnosed with inflammatory bowel disease between 2018 and 2023, who were followed up at the Department of Pediatric Gastroenterology, Hepatology, and Nutrition at University of Health Sciences, Izmir Dr. Behçet Uz Children’s Hospital. The study continued with 70 patients diagnosed with ulcerative colitis, according to the Porto criteria. Eight patients were excluded from the study due to insufficient data and inability to follow up. The retrospective, single-center, cohort study continued with 62 patients.

Demographic data included age and gender at diagnosis, anthropometric measurements such as body mass index (BMI), weight-for-age, and height-for-age. Laboratory parameters recorded were complete blood count, total protein, albumin, C-reactive protein (CRP), sedimentation rate, immunoglobulin G (IgG), immunoglobulin M (IgM), immunoglobulin A (IgA), IgG subclasses, vitamin D, B12, folic acid, and ferritin levels.

Considering the WHO’s references, BMI, height and weight values <−2 Z-score, between −2 to +2, and ≥+2 were categorized as low, sufficient, and high, respectively. https://www.who.int/tools/child-growth-standards/standards (accessed on 21 December 2025).

WHO recommendations were used to determine patients’ anemia status. https://www.who.int/publications/i/item/9789240088542 (accessed on 21 December 2025).

According to the PUCAI score, values <10 were classified as remission, 10–34 as mild disease activity, 35–64 as moderate disease activity, and ≥65 as severe disease activity [11].

The modified Mayo endoscopy subscore (MMES) was used to assess the endoscopic activity of the disease [23]. The spread of the disease was also classified into four groups according to the Paris classification: proctitis (E1), distal colitis extending from the rectum to the splenic flexure (E2), diffuse colitis extending to the hepatic flexure (E3), and pancolitis extending proximal to the hepatic flexure (E4) [10].

A flare-up during or within 1 month after stopping steroid treatment was considered steroid dependence [18,19,20,24]. Detection of PUCAI >10 during follow-up outside this period was considered as flare-up and relapse [16,18,19,20]. Patients were treated according to the recommendations of the ESPGHAN guidelines [25].

Patients were followed for a period of 2 years. Study endpoints included the development of steroid dependence and relapse during follow-up. Steroid-free remission was defined as the maintenance of a PUCAI score <10 throughout the entire 2-year follow-up period.

The associations between age at diagnosis, sex, anthropometric measurements, laboratory parameters, PUCAI score, endoscopic disease activity, and the outcomes of steroid dependence, relapse, and steroid-free remission were evaluated.

The statistical analysis was conducted using the SPSS v26.0 (IBM, Chicago, IL, USA) software package for Windows. The Shapiro–Wilk test was used to determine whether the parameters evaluated in the study showed a normal distribution. Nominal and ordinal variables were compared with the chi-square test of independence. The chi-square test of independence was employed to assess the relationship between nominal and ordinal variables. In the event that the comparison of numerical variables was parametric according to the distribution, a paired-sample *t*-test was employed; conversely, if nonparametric, Wilcoxon’s two-sample test was utilized. In the case of comparisons between more than two independent groups, the Kruskal–Wallis test was employed. A logistic regression analysis was conducted for laboratory parameters. To determine the accuracy of laboratory markers for prognostic tests, receiver operating characteristic (ROC) curves were drawn by plotting sensitivity against 1-specificity. Overall accuracy of the marker in predicting the respective disease outcome was represented by area under the ROC curve (AUC) with 95% CI. The best cut-off point was calculated using the Youden-Index, defined as the maximum sum of sensitivity and specificity.

## 3. Results

### 3.1. Participant Characteristics

The study included 62 patients, 32 of whom were female and 30 of whom were male (Table 1). The median age of the patients was 14 years (IQR, 25th–75th percentile: 12–16). Four patients were diagnosed with very early onset and seven with early-onset inflammatory bowel disease (IBD). During follow-up, 32 patients (48.4%) experienced relapse, and 12 (19.4%) developed steroid dependence. The median follow-up duration of the patients was 29 months (IQR, 25th–75th percentile: 24–40). The mean duration of the relapse period for patients was 6.79 ± 2.01 months. Twenty patients (32.3%) did not experience relapse during the 2-year follow-up period. Two patients (3.0%) were primarily unresponsive to steroid treatment. 10 patients (16.1%) were steroid-dependent due to flare-up under steroid treatment. Of the patients who relapsed, 23 (76.6%) had pancolitis, 3 (10.0%) had extensive colitis, and 4 (13.4%) had distal colitis. 100% of steroid-dependent patients had pancolitis.

### 3.2. Baseline Participant Laboratory Parameters

Anemia was present in 77.4% of patients, low iron levels in 86.4%, and low ferritin levels in 65.2%. Vitamin D deficiency was present in 66.1%, B12 deficiency in 4%, and folic acid deficiency in 18.4%. At diagnosis, elevated ESR was observed in 86.2% of patients and elevated CRP in 15.3% (Table 2).

### 3.3. The Relationship Between Demographic, Anthropometric, and Laboratory Parameters and Relapse

There was no association between gender and age and relapse (*p =* 0.90, *p =* 0.85, respectively). The early-onset or very early-onset IBD patient group did not differ from other age groups in terms of relapse and steroid dependency (*p =* 0.99). There was no association between weight-for-age and height-for-age Z-scores (*p =* 0.11, *p =* 0.74, respectively). There was a significant association between BMI Z-score and relapse (*p =* 0.05).

In the univariate regression analysis, a relationship was identified between white blood cell count, neutrophil count, eosinophil count, total protein, vitamin D levels, and relapse (*p =* 0.01, *p =* 0.01, *p =* 0.04, *p =* 0.04, *p =* 0.03, respectively) (Table 3 and Table 4).

IgM levels at diagnosis were higher in patients who did not relapse (*p =* 0.04). In the univariate regression analysis, there was an inverse correlation between IgM levels and relapse (*p* < 0.01). No association was found between IgG levels and relapse (*p =* 0.49).

In the multivariable logistic regression analysis, higher PUCAI scores and MMES were independently associated with relapse (*p =* 0.03, *p* < 0.01, respectively). Conversely, higher IgM levels were found to be inversely associated with relapse (*p* < 0.01) (Table 5). The final model demonstrated good explanatory power with a Nagelkerke R^2^ of 0.681 and an overall classification accuracy of 86.9%.

### 3.4. The Relationship Between Demographic, Anthropometric, and Laboratory Parameters and Steroid Dependency

No significant relationship was found between steroid dependency and gender or age (*p* = 0.78 and *p* = 0.33, respectively). There was no difference in steroid dependency between the early-onset and very early-onset inflammatory bowel disease (IBD) patient groups and other age groups (*p =* 0.55).

There was no relationship between steroid dependency and weight-for-age, height-for-age and BMI Z-scores (*p =* 0.95, *p =* 0.34, *p =* 0.85, respectively).

In the univariate regression analysis, a relationship was identified between steroid dependency and hemoglobin, hematocrit, white blood cell count, neutrophil count, platelet count, and albumin levels (*p =* 0.01, *p* < 0.01, *p* < 0.01, *p =* 0.01, *p* < 0.01, *p =* 0.03, respectively) (Table 6).

**Figure 1:**
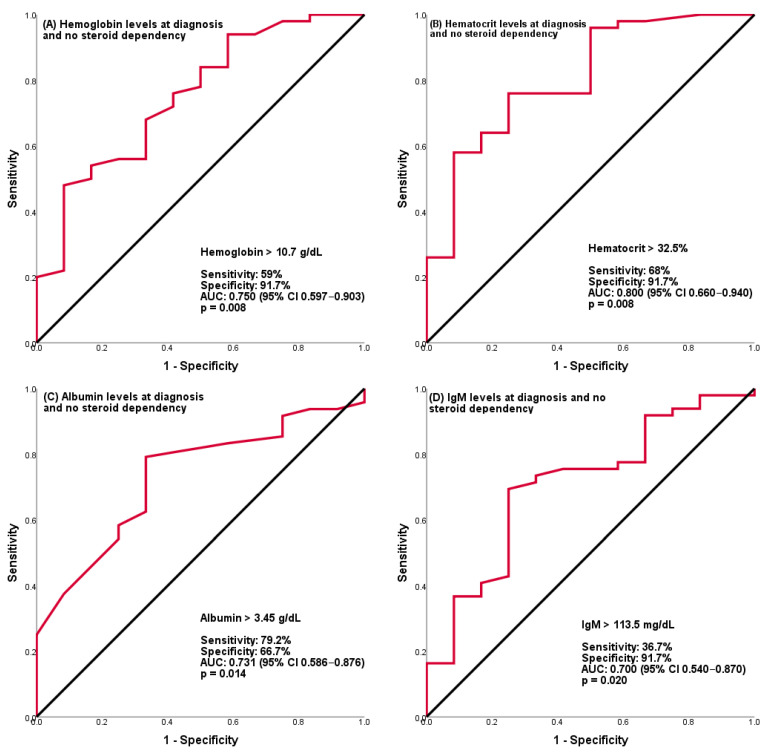
Prediction of no steroid dependency in ulcerative colitis patients. Shown are ROC analyses for prediction of no steroid dependency. Abbreviations: AUC, Area under the ROC curve; CI, Confidence Interval; IgM, Immunoglobulin M.

IgG levels at diagnosis were significantly elevated in patients with steroid dependency (*p* < 0.01). A significant relationship was found between steroid dependency and IgG levels (*p =* 0.02). In the univariate regression analysis, IgM levels were inversely correlated with steroid dependency (*p =* 0.03) (Figure 1).

Extensive and distal patients had higher IgM levels than the pancolitis group (*p =* 0.02).

### 3.5. The Relationship Between MMES, PUCAI Score and Relapse and Steroid Dependency

**Figure 2:**
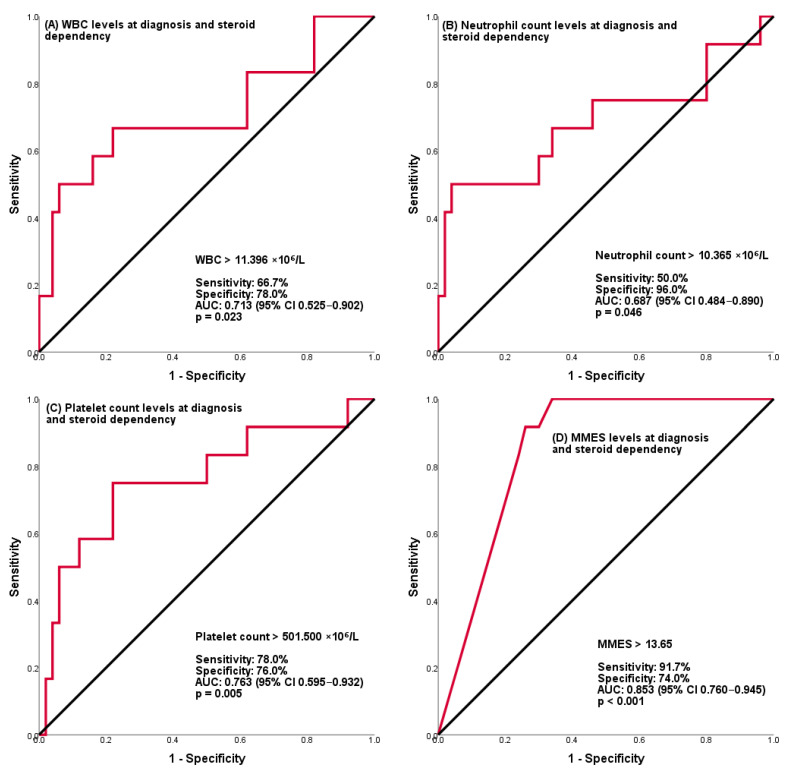
Prediction of steroid dependency in ulcerative colitis patients. Shown are ROC analyses for prediction of steroid dependency. Abbreviations: AUC, Area under the ROC curve; CI, Confidence interval; MMES, Mayo endoscopic subscore; WBC, White blood cell count.

MMES was significantly higher in groups with relapse and steroid dependence (*p =* 0.04, *p* < 0.01) (Figure 2) (Table 7).

Significantly high MMES was detected in patients with pancolitis at diagnosis (*p* < 0.01). A significant relationship was detected between steroid dependency and high MMES in patients with pancolitis at diagnosis (*p =* 0.03).

MMES was found to be significant in predicting steroid dependency in patients with pancolitis (AUC: 0.75, 95% CI: [0.60–0.90], *p =* 0.01).

Odds ratios (ORs) and 95% confidence intervals (CIs) were obtained from univariate logistic regression analyses. Nagelkerke R^2^ values indicate the explained variance of each model. A *p* value < 0.05 was considered statistically significant.

A significant relationship was found between PUCAI score and relapse, while no significant relationship was found between PUCAI score and steroid dependency (*p =* 0.01, *p =* 0.06, respectively).

In the multivariable logistic regression analysis evaluating predictors of steroid dependence, higher modified Mayo endoscopic scores (MMES) were independently associated with steroid dependence (adjusted OR 2.58, 95% CI 1.11–6.04; *p* = 0.028). Platelet count also remained significantly associated with steroid dependence (adjusted OR 1.00, 95% CI 1.00–1.00; *p* = 0.017). In contrast, IgM levels showed an inverse association with steroid dependence (adjusted OR 0.97, 95% CI 0.94–1.00; *p* = 0.049). The final model demonstrated good explanatory power, with a Nagelkerke R^2^ of 0.624 and an overall classification accuracy of 90.2% (Table 8).

### 3.6. The Relationship Between Administered Treatment and Relapse and Steroid Dependence

All patients were started on steroids and mesalazine at diagnosis. Fifty-two patients received oral steroid therapy and 10 patients received IV steroid therapy. The average initial steroid dose for patients was 43.2 mg/day. The average mesalazine dose for patients was 60 mg/kg/day.

In the univariate regression analysis, no association was found between the administered steroid dose and relapse or steroid dependency (*p* = 0.56 and *p* = 0.88, respectively). Similarly, no association was found between relapse and steroid dependency with mesalazine doses (*p* = 0.09, *p* = 0.24, respectively).

Azathioprine treatment was initiated for 42 patients during the second month. The average azathioprine dosage was 1.58 mg/kg/day. Two patients were primary steroid resistant, and six patients relapsed within two months, so they transitioned to the next level of treatment before azathioprine therapy could begin. Twelve patients with mild disease were treated with mesalazine alone.

There was no difference in relapse development between patients receiving azathioprine treatment (*p =* 0.72). Similarly, no difference was found between patients receiving 2 mg/kg/day or higher doses of azathioprine and those receiving lower doses (*p =* 0.28).

## 4. Discussion

Studies evaluating various laboratory parameters, endoscopic and colonoscopic findings, anthropometric measurements, and microbiome status have identified predictive criteria for steroid dependency in pediatric ulcerative colitis. However, unlike in Crohn’s disease, the clinical situations in which anti-TNF therapy can be used as a first-line treatment for ulcerative colitis have not yet been clearly determined [14,16,18,26].

The study found no significant differences between male and female pediatric ulcerative colitis patients with regard to steroid dependency and relapse. Despite the findings of Claßen et al., which indicated a correlation between older age and the utilization of biological agents, no such correlation was identified in the present study or in other extant literature [17,18,22,27,28,29]. Many publications demonstrate that growth retardation in Crohn’s disease patients is associated with poor prognosis and is an indication for anti-TNF treatment [14,16,22,28]. However, the same is not true for ulcerative colitis. Consistent with our study data, no relationship was found between height, body weight, and BMI Z-scores and disease prognosis [17,18,22,27,28,29].

In the assessment of the correlation between laboratory parameters and disease prognosis, a number of studies have identified anemia, hypoalbuminemia, thrombocytosis, and peripheral eosinophilia as potential indicators of steroid dependency and of the necessity for biological agent treatment. In our study, hemoglobin and hematocrit levels were found to be significantly low in the steroid-dependent patient group; however, hemoglobin and hematocrit levels above 10.7 g/dL and 32.5%, respectively, were found to be significant in terms of not developing steroid dependency with AUC: 0.75, 50% sens., 91.7% spec., and AUC: 0.80, 58% sens., 91.7% spec., respectively. The study by Gasparetto et al. found an association between low hemoglobin levels and severity of disease [30]. In the study by Hoeve et al., hemoglobin levels were found to be significantly low in patients requiring rescue therapy while receiving azathioprine treatment [28]. In contrast, Claßen et al. reported that hemoglobin and hematocrit levels were unrelated to steroid dependence. However, a relationship was identified between hematocrit levels and the use of biological agents [18].

In the study, platelet counts above 501,500 × 10^6^/L were found to be predictive of steroid dependency, with AUC: 0.76, 75% sens., and 78% specificity. A review of the literature did not show any correlation between platelet count and steroid dependency or severity of disease [18,27,28,30]. However, it was recently shown in tissue and serum samples that platelet count may be associated with histological activity and may predict disease severity [31,32,33]. Some studies have identified a significant association between hypoalbuminemia and severe disease progression in looking at prognostic factors [27,30,34].

The findings of the present study indicate that albumin levels were lower in the steroid-dependent group. However, albumin levels above 3.45 g/dL were found to be significant in terms of not developing steroid dependence, with AUC: 0.73, 79.2% sens. and 66.7% specificity. In our study, WBC above 11,395 × 10^6^/L showed AUC: 0.71, 66.7% spec., and 78% sens., neutrophil count above 10,365 × 10^6^/L showed AUC: 0.68, 50% sens., and 96% spec. and was found to be significant in predicting steroid dependence. In a recent study by Aziz et al. involving 580 pediatric ulcerative colitis patients, the neutrophil/lymphocyte ratio and elevated WBC were found to be associated with endoscopic activity severity [35]. In agreement with others, our work found no significant relationship between acute phase markers and steroid dependency [25,29].

Our results showed a significant association between pancolitis, PUCAI score, and MMES with severe disease. Claßen et al. found that a PUCAI score above 65 was associated with steroid dependence [18]. Nambu et al. showed that the need for treatment with biological agents increased among patients with a PUCAI score above 65 [26]. Gasparetto et al. reported that patients presenting with pancolitis had a more severe disease course [30]. Our own results indicated that an MMES score above 13.65 was associated with steroid dependency, with AUC: 0.85, 91.7% sens., and 74% specificity. The findings of Schechter et al. indicated that low hemoglobin, endoscopic disease activity, and albumin levels are associated with acute severe colitis [34]. And Yoon et al. indicated an association between high Mayo endoscopy scores at the time of diagnosis and steroid dependency in a group of adult patients [36]. Torres et al. reported in their study with an adult patient group that the extent of the disease is associated with disease severity [37].

There is limited information about the relationship between immunoglobulin levels and disease prognosis. Our work found IgM levels above 84.5 mg/dL to be significant in terms of not developing steroid dependence, with AUC: 0.70, 69.4% sens. and 75% specificity. IgM levels were significantly higher in patients with extensive and distal colitis, compared to patients with pancolitis. Moreover, this was significant in predicting steroid-free remission with AUC: 0.76, 74.2% sens., 80% spec. above 99.5. Song et al. examined 608 adults with Crohn’s disease, and found there was an inverse correlation between ileal disease severity and IgM levels [38]. Various studies have shown that IgM levels vary due to dysbiosis or genetic causes [39,40,41]. Preisker et al. reported a significant increase in intestinal IgM^+^ B cells in Crohn’s disease patients in remission [42]. Although data are scant on this, it has been suggested that IgM levels may be associated with mucosal health. It should be noted that IgM levels were analyzed as a continuous variable, and the observed associations reflect relative differences within the cohort rather than age-adjusted immunoglobulin deficiency.

From a methodological standpoint, the findings of this study highlight the need for prospective and multicenter investigations to further explore the prognostic value of combined clinical, laboratory, and endoscopic parameters in pediatric ulcerative colitis. Such studies may help determine whether these markers can be integrated into structured risk assessment models, while accounting for disease heterogeneity and treatment variability.

## 5. Conclusions

Our report makes use of data analyzed in a retrospective manner, and so because the work did not have prospective planning, it was not possible to evaluate patient compliance with treatment and assess patients based on histological response.

We conclude that, as with Crohn’s disease, an algorithm or a specific scoring system for ulcerative colitis is needed for the use of anti-TNF drugs as first-line treatment in pediatric ulcerative colitis. The initial severity of the disease appears to be the most important risk factor in terms of steroid dependence. Based on our study and the literature data, a scoring system incorporating parameters such as hemoglobin, hematocrit, WBC, albumin, platelet, disease involvement type, initial PUCAI score, and MMES would be prudent to adopt. While the study data demonstrate the significance of IgM levels, further research is necessary to reach more precise conclusions. We believe more definitive results will be obtained with larger patient series and the use of prospective planning.

## Figures and Tables

**Table 1:** Baseline descriptive statistics of participant characteristics.

		Patients (%), *n* = 62
Sex	Female	32 (51.6%)
	Male	30 (48.4%)
Age	Mean (SD)	13.2 (3.22)
	3–6 yr.	4 (6.5%)
	6–10 yr.	8 (12.9%)
	10–17 yr.	40 (80.6%)
Weight SDS	Mean (SD)	−0.45 (1.66)
Height SDS	Mean (SD)	0.21 (1.08)
BMI SDS	Mean (SD)	−0.83 (1.78)
PUCAI	Mean (SD)	50.8 (17.21)
	10–34	8 (12.9%)
	35–64	35 (56.5%)
	>65	19 (30.6%)
Disease extent	Proctitis (E1)	5 (8.1%)
	Left side colitis (E2)	10 (16.1%)
	Extensive colitis (E3)	8 (12.9%)
	Pancolitis (E4)	39 (62.9%)
Upper GI involvement	Yes	27 (61.4%)
	No	17 (38.6%)
Upper GI involvement	Esophagitis	18 (66.6%)
	Gastritis	4 (14.9%)
	Duodenitis	2 (7.4%)
	Gastritis and Duodenitis	3 (11.1%)
Relapse	Yes	30 (48.4%)
	No	32 (51.6%)
Steroid dependency	Yes	12 (19.4%)
	No	50 (80.6%)
Relapse (+)	E1	1 (3.3%)
	E2	3 (10%)
	E3	3 (10%)
	E4	23 (76.7%)
Steroid dependent	E4	12 (100%)

Abbreviations: SD, Standard deviation; SDS, Standard deviation score; BMI, Body mass index; PUCAI, Pediatric ulcerative colitis activity index; GI: Gastrointestinal.

**Table 2:** Descriptive statistics of predictor parameters.

	Patients *n* (%)	Mean (SD)	Minimum (Min.) Maximum (Max.)
Hemoglobin (g/dL)	62 (100%)	10.2 (2.4)	5.8–15.1
Hematocrit (%)	62 (100%)	32.3 (6.1)	17.7–44.5
White blood cell count (×10^6^/L)	62 (100%)	10,587 (3602)	5520–20,020
Neutrophil count (×10^6^/L)	62 (100%)	6816 (2875)	2390–15,440
Lymphocyte count (×10^6^/L)	62 (100%)	2491 (917)	970–5110
Platelet count (×10^6^/L)	62 (100%)	447,642 (154,566)	180,000–846,000
Albumin (g/dL)	60 (96.8%)	3.8 (0.7)	2.1–4.9
CRP (mg/dL)	59 (95.1%)	0.92 (1.80)	0.02–10.06
ESR (mm/h)	58 (93.5%)	39 (18)	8–82
IgG (mg/dL)	62 (100%)	1267 (328)	745–2030
IgM (mg/dL)	61 (98.4%)	103 (44)	32–249
IgA (mg/dL)	59 (95.1%)	173 (74)	25–374
IgG1 (g/L)	29 (46.7%)	1023 (254)	559–1620
IgG2 (g/L)	29 (46.7%)	248 (103)	53–459
IgG3 (g/L)	29 (46.7%)	57 (32)	23–126
IgG4 (g/L)	29 (46.7%)	73 (59)	6–260
Vitamin D (µg/L)	41 (66.1%)	14.42 (5.82)	4.6–24.9
B12 (ng/L)	50 (80.6%)	510 (278)	168–1684
Folic acid (µg/L)	38 (61.2%)	6.5 (3.0)	2.4–14.3
Ferritin (µg/L)	46 (74.2%)	11.2 (10.0)	1.0–44.0

Abbreviations: SD, Standard deviation; CRP, C-reactive protein; ESR, Erythrocyte sedimentation rate; IgG, Immunoglobulin G; IgM, Immunoglobulin M; IgA, Immunoglobulin A.

**Table 3:** Predictor parameters of disease relapse.

	Relapse				
	(−)		(+)		*p* Value
	Mean (SD)	Min.–Max.	Mean (SD)	Min.–Max.	
BMI SDS	−0.41 (1.65)	−3.77–2.11	−1.29 (1.84)	−5.53–1.24	0.05
White blood cell count (×10^6^/L)	9184 (2694)	5450–16,360	11,419 (4011)	5530–20,020	<0.01
Neutrophil count (×10^6^/L)	5642 (2097)	2390–10,870	7548 (3122)	3050–15,440	0.02
Eosinophil count (×10^6^/L)	263 (209)	20–830	433 (394)	10–1190	<0.01
Total Protein (g/dL)	7.0 (0.9)	5–9	6.5 (1.0)	4.2–7.8	0.03
Vitamin D (µg/L)	16.50 (5.25)	6.8–24.9	12.62 (5.79)	4.6–22.6	0.03
IgM (mg/dL)	119 (48)	30–249	81 (27)	32–138	<0.01
MMES	10.5 (2.7)	4–15	13.8 (1.9)	8–15	<0.01
PUCAI	45.47 (16.03)	20–75	56.67 (16.78)	30–80	<0.01

Abbreviations: SD, Standard deviation; BMI, Body mass index; SDS, Standard deviation score; IgM, Immunoglobulin M; MMES, Modified Mayo endoscopic subscore; PUCAI, Pediatric ulcerative colitis activity index.

**Table 4:** Univariate logistic regression analysis of predictors of relapse.

	OR (95% CI)	Nagelkerke R^2^	*p* Value
BMI SDS	0.74 (—)	0.081	0.05
White blood cell count (×10^6^/L)	1.00 (1.00–1.00)	0.133	0.01
Neutrophil count (×10^6^/L)	1.00 (1.00–1.001)	0.158	0.01
Eosinophil count (×10^6^/L)	1.002 (1.000–1.004)	0.095	0.04
Total protein (g/dL)	0.54 (0.30–0.99)	0.099	0.04
Vitamin D (µg/L)	0.88 (0.78–0.99)	0.149	0.03
IgM (mg/dL)	0.97 (0.95–0.99)	0.275	<0.01
MMES	1.81 (1.35–2.41)	0.450	<0.01
PUCAI	1.04 (1.01–1.08)	0.140	0.01

Odds ratios (ORs) and 95% confidence intervals (CIs) were obtained from univariate logistic regression analyses. Nagelkerke R^2^ values indicate the explained variance of each model. A *p* value < 0.05 was considered statistically significant. Abbreviations: BMI, Body mass index; SDS, Standard deviation score; IgM, Immunoglobulin M; MMES, Modified Mayo endoscopic subscore; PUCAI, Pediatric ulcerative colitis activity index.

**Table 5:** Multivariable logistic regression analysis of predictors of relapse.

	Adjusted OR (95% CI)	*p* Value
Neutrophil count (×10^6^/L)	1.00 (1.00–1.001)	0.08
IgM (mg/dL)	0.97 (0.94–0.99)	<0.01
MMES (Modified Mayo score)	1.88 (1.25–2.83)	<0.01
PUCAI	1.05 (1.00–1.11)	0.03

Abbreviations: OR, Odds ratio; CI, Confidence Interval; IgM, Immunoglobulin M; MMES, Modified Mayo endoscopic subscore; PUCAI, Pediatric ulcerative colitis activity index. Adjusted odds ratios (ORs) and 95% confidence intervals (CIs) were obtained from multivariable logistic regression analysis. A *p* value < 0.05 was considered statistically significant.

**Table 6:** Predictor parameters of steroid dependency.

	Steroid Dependency				
	(−)		(+)		*p* Value
	Mean (SD)	Min.-Max.	Mean (SD)	Min.-Max.	
Hemoglobin (g/dL)	10.5 (2.3)	6.7–15.1	8.5 (2)	5.8–12.7	<0.01
Hematocrit (%)	33.5 (5.3)	23.8–44.5	27.2 (5.3)	17.7–37.3	<0.01
White blood cell count (×10^6^/L)	9600 (2956)	5450–17,320	13,040 (4536)	6600–20,020	<0.01
Neutrophil count (×0^6^/L)	6079 (2218)	2390–11,480	8588 (3971)	3050–15,440	<0.01
Platelet count (×10^6^/L)	420,400 (129,132)	180,000–846,000	564,667 (163,552)	278,000–830,000	<0.01
Albumin (g/dL)	3.9 (0.7)	2.1–4.9	3.4 (0.6)	2.4–4.3	0.02
IgG (mg/dL)	1203 (276)	838–1950	1453 (447)	745–2030	0.01
IgM (mg/dL)	106 (44)	30–249	76 (32)	32–138	0.03
MMES	11.4 (2.8)	4–15	14.8 (0.6)	13–15	<0.01
PUCAI	48.9 (16.79)	25–80	59.17 (17.17)	20–80	**0.06**

Abbreviations: SD, Standard deviation; IgG, Immunoglobulin G; IgM, Immunoglobulin M; MMES, Modified Mayo endoscopic subscore; PUCAI, Pediatric ulcerative colitis activity index.

**Table 7:** Univariate logistic regression analysis of predictors of steroid dependence.

	OR (95% CI)	Nagelkerke R^2^	*p* Value
Hemoglobin (g/dL)	0.62 (0.43–0.90)	0.201	0.012
Hematocrit (%)	0.79 (0.67–0.92)	0.301	0.003
White blood cell count (×10^6^/L)	1.00 (1.00–1.00)	0.211	0.006
Neutrophil count (×10^6^/L)	1.00 (1.00–1.001)	0.181	0.011
Platelet count (×10^6^/L)	1.00 (1.00–1.00)	0.219	0.006
Albumin (g/dL)	0.37 (0.15–0.91)	0.126	0.030
IgG (mg/dL)	1.00 (1.00–1.004)	0.133	0.024
IgM (mg/dL)	0.98 (0.96–1.00)	0.140	0.036
MMES	2.97 (1.29–6.82)	0.448	0.010

Abbreviations: OR, Odds ratio; CI, Confidence Interval; IgG, Immunoglobulin G; IgM, Immunoglobulin M; MMES, Modified Mayo endoscopic subscore.

**Table 8:** Multivariable logistic regression analysis of predictors of steroid dependence.

	Adjusted OR (95% CI)	*p* Value
Platelet count (×10^6^/L*)*	1.00 (1.00–1.00)	0.01
IgM (mg/dL)	0.97 (0.94–1.00)	0.04
MMES	2.58 (1.11–6.04)	0.02

Abbreviations: OR, Odds ratio; CI, Confidence Interval; IgM, Immunoglobulin M; MMES, Modified Mayo endoscopic subscore. Adjusted odds ratios (ORs) and 95% confidence intervals (CIs) were obtained from multivariable logistic regression analysis. A *p* value < 0.05 was considered statistically significant.

## Data Availability

The raw data supporting the conclusions of this article will be made available by the authors on request.

## References

[1] Turner D., Levine A., Escher J.C., Griffiths A.M., Russell R.K., Dignass A., Dias J.A., Bronsky J., Braegger C.P., Cucchiara S. (2012). Management of Pediatric Ulcerative Colitis. J. Pediatr. Gastroenterol. Nutr..

[2] Wang Y., Pan C.-W., Huang Y., Zheng X., Li S., He M., Hashash J.G., Farraye F.A., Ehrlich A.C. (2025). Global Epidemiology and Geographic Variations of Pediatric-Onset Inflammatory Bowel Disease: A Comprehensive Analysis of the Global Burden of Disease Study 1990 to 2019. Inflamm. Bowel Dis..

[3] Benchimol E.I., Fortinsky K.J., Gozdyra P., Van den Heuvel M., Van Limbergen J., Griffiths A.M. (2011). Epidemiology of Pediatric Inflammatory Bowel Disease: A Systematic Review of International Trends. Inflamm. Bowel Dis..

[4] Kudo T., Shimizu T. (2023). Mucosal Immune Systems of Pediatric Inflammatory Bowel Disease: A Review. Pediatr. Int..

[5] Mizoguchi A., Bhan A.K., Baumgart D.C. (2012). Immunobiology of B Cells in Inflammatory Bowel Disease. Crohn’s Disease and Ulcerative Colitis: From Epidemiology and Immunobiology to a Rational Diagnostic and Therapeutic Approach.

[6] Bhalla A., Shahi A., Maity M., Safa F.N.U., Srividya V., Clementina R., Anugu G.R., Younas S., Bhalla A., Shahi A. (2025). Inflammatory Bowel Disease in Children: Current Diagnosis and Treatment Strategies. Cureus.

[7] Rogler G., Singh A., Kavanaugh A., Rubin D.T. (2021). Extraintestinal Manifestations of Inflammatory Bowel Disease: Current Concepts, Treatment, and Implications for Disease Management. Gastroenterology.

[8] Levine A., Koletzko S., Turner D., Escher J.C., Cucchiara S., de Ridder L., Kolho K.-L., Veres G., Russell R.K., Paerregaard A. (2014). ESPGHAN Revised Porto Criteria for the Diagnosis of Inflammatory Bowel Disease in Children and Adolescents. J. Pediatr. Gastroenterol. Nutr..

[9] Silverberg M.S., Satsangi J., Ahmad T., Arnott I.D.R., Bernstein C.N., Brant S.R., Caprilli R., Colombel J.-F., Gasche C., Geboes K. (2005). Toward an Integrated Clinical, Molecular and Serological Classification of Inflammatory Bowel Disease: Report of a Working Party of the 2005 Montreal World Congress of Gastroenterology. Can. J. Gastroenterol. J. Can. Gastroenterol..

[10] Levine A., Griffiths A., Markowitz J., Wilson D.C., Turner D., Russell R.K., Fell J., Ruemmele F.M., Walters T., Sherlock M. (2011). Pediatric Modification of the Montreal Classification for Inflammatory Bowel Disease: The Paris Classification. Inflamm. Bowel Dis..

[11] Turner D., Hyams J., Markowitz J., Lerer T., Mack D.R., Evans J., Pfefferkorn M., Rosh J., Kay M., Crandall W. (2009). Appraisal of the Pediatric Ulcerative Colitis Activity Index (PUCAI). Inflamm. Bowel Dis..

[12] Hyams J.S., Davis S., Mack D.R., Boyle B., Griffiths A.M., LeLeiko N.S., Sauer C.G., Keljo D.J., Markowitz J., Baker S.S. (2017). Factors Associated with Early Outcomes Following Standardised Therapy in Children with Ulcerative Colitis (PROTECT): A Multicentre Inception Cohort Study. Lancet Gastroenterol. Hepatol..

[13] Turner D., Seow C.H., Greenberg G.R., Griffiths A.M., Silverberg M.S., Steinhart A.H. (2009). A Systematic Prospective Comparison of Noninvasive Disease Activity Indices in Ulcerative Colitis. Clin. Gastroenterol. Hepatol..

[14] Wine E., Aloi M., Van Biervliet S., Bronsky J., Di Carpi J.M., Gasparetto M., Gianolio L., Gordon H., Hojsak I., Hudson A.S. (2025). Management of Paediatric Ulcerative Colitis, Part 1: Ambulatory Care—An Updated Evidence-based Consensus Guideline from the European Society of Paediatric Gastroenterology, Hepatology and Nutrition and the European Crohn’s and Colitis Organisation. J. Pediatr. Gastroenterol. Nutr..

[15] Yerushalmy-Feler A., Olbjorn C., Kolho K.-L., Aloi M., Musto F., Martin-de-Carpi J., Lozano-Ruf A., Yogev D., Matar M., Scarallo L. (2024). Dual Biologic or Small Molecule Therapy in Refractory Pediatric Inflammatory Bowel Disease (DOUBLE-PIBD): A Multicenter Study from the Pediatric IBD Porto Group of ESPGHAN. Inflamm. Bowel Dis..

[16] van Rheenen P.F., Aloi M., Assa A., Bronsky J., Escher J.C., Fagerberg U.L., Gasparetto M., Gerasimidis K., Griffiths A., Henderson P. (2021). The Medical Management of Paediatric Crohn’s Disease: An ECCO-ESPGHAN Guideline Update. J. Crohn’s Colitis.

[17] Hyams J.S., Thomas S.D., Gotman N., Haberman Y., Karns R., Schirmer M., Mo A., Mack D.R., Boyle B., Griffiths A.M. (2019). Clinical and Biological Predictors of Response to Standardised Paediatric Colitis Therapy: A Multicentre Inception Cohort Study. Lancet Lond. Engl..

[18] Claßen M., Schiller B., Däbritz J. (2024). CEDATA-GPGE Study Group Predicting Complications in Paediatric Ulcerative Colitis: A Longitudinal Multicentre Cohort Study. Aliment. Pharmacol. Ther..

[19] Orlanski-Meyer E., Aardoom M., Ricciuto A., Navon D., Carman N., Aloi M., Bronsky J., Däbritz J., Dubinsky M., Hussey S. (2021). Predicting Outcomes in Pediatric Ulcerative Colitis for Management Optimization: Systematic Review and Consensus Statements From the Pediatric Inflammatory Bowel Disease-Ahead Program. Gastroenterology.

[20] Aloi M., D’Arcangelo G., Pofi F., Vassallo F., Rizzo V., Nuti F., Di Nardo G., Pierdomenico M., Viola F., Cucchiara S. (2013). Presenting Features and Disease Course of Pediatric Ulcerative Colitis. J. Crohn’s Colitis.

[21] Nambu R., Arai K., Kudo T., Murakoshi T., Kunisaki R., Mizuochi T., Kato S., Kumagai H., Inoue M., Ishige T. (2023). Clinical Outcome of Ulcerative Colitis with Severe Onset in Children: A Multicenter Prospective Cohort Study. J. Gastroenterol..

[22] Tanpowpong P., Treepongkaruna S., Huang J.G., Chew K.S., Mercado K.S.C., Reodica A., Rajindrajith S., Hathagoda W., Wong Y.K.Y., Lee W.S. (2025). Outcome of Pediatric Inflammatory Bowel Disease in Asian Children: A Multinational 1-Year Follow-up Study. Clin. Exp. Pediatr..

[23] Lobatón T., Bessissow T., De Hertogh G., Lemmens B., Maedler C., Van Assche G., Vermeire S., Bisschops R., Rutgeerts P., Bitton A. (2015). The Modified Mayo Endoscopic Score (MMES): A New Index for the Assessment of Extension and Severity of Endoscopic Activity in Ulcerative Colitis Patients. J. Crohn’s Colitis.

[24] Munkholm P., Langholz E., Davidsen M., Binder V. (1994). Frequency of Glucocorticoid Resistance and Dependency in Crohn’s Disease. Gut.

[25] Turner D., Ruemmele F.M., Orlanski-Meyer E., Griffiths A.M., de Carpi J.M., Bronsky J., Veres G., Aloi M., Strisciuglio C., Braegger C.P. (2018). Management of Paediatric Ulcerative Colitis, Part 1: Ambulatory Care-An Evidence-Based Guideline from European Crohn’s and Colitis Organization and European Society of Paediatric Gastroenterology, Hepatology and Nutrition. J. Pediatr. Gastroenterol. Nutr..

[26] Elhag D.A., Kumar M., Saadaoui M., Akobeng A.K., Al-Mudahka F., Elawad M., Al Khodor S. (2022). Inflammatory Bowel Disease Treatments and Predictive Biomarkers of Therapeutic Response. Int. J. Mol. Sci..

[27] Tanpowpong P., Jitwongwai S., Kijmassuwan T., Sriphongphankul H., Osatakul S., Damrongmanee A., Ukarapol N., Treepongkaruna S. (2024). Multicenter Registry of Pediatric Inflammatory Bowel Disease from a Developing Country. BMC Pediatr..

[28] van Hoeve K., Hoffman I., D’Hoore A., Ferrante M., Vermeire S. (2020). Long-Term Outcome of Immunomodulator Use in Pediatric Patients with Inflammatory Bowel Disease. Dig. Liver Dis..

[29] Coughlan A., Wylde R., Lafferty L., Quinn S., Broderick A., Bourke B., Hussey S. (2017). DOCHAS Study A Rising Incidence and Poorer Male Outcomes Characterise Early Onset Paediatric Inflammatory Bowel Disease. Aliment. Pharmacol. Ther..

[30] Gasparetto M., Wong-Spracklen V., Torrente F., Howell K., Brennan M., Noble-Jamieson G., Heuschkel R., Zilbauer M. (2018). Early Treatment Response Predicts Outcome in Paediatric Ulcerative Colitis: GASTROENTEROLOGY: INFLAMMATORY BOWEL DISEASE. J. Pediatr. Gastroenterol. Nutr..

[31] Schiller B., Wirthgen E., Weber F., Schiller S., Radke M., Claßen M., Däbritz J. (2024). Fecal Calprotectin and Platelet Count Predict Histologic Disease Activity in Pediatric Ulcerative Colitis: Results from a Projection-Predictive Feature Selection. Eur. J. Pediatr..

[32] Janker L., Schuster D., Bortel P., Hagn G., Meier-Menches S.M., Mohr T., Mader J.C., Slany A., Bileck A., Brunmair J. (2023). Multiomics-Empowered Deep Phenotyping of Ulcerative Colitis Identifies Biomarker Signatures Reporting Functional Remission States. J. Crohn’s Colitis.

[33] Nakarai A., Kato J., Hiraoka S., Takashima S., Inokuchi T., Takahara M., Sugihara Y., Harada K., Okada H. (2018). An Elevated Platelet Count Increases the Risk of Relapse in Ulcerative Colitis Patients with Mucosal Healing. Gut Liver.

[34] Schechter A., Griffiths C., Gana J.C., Shaoul R., Shamir R., Shteyer E., Bdolah-Abram T., Ledder O., Turner D. (2015). Early Endoscopic, Laboratory and Clinical Predictors of Poor Disease Course in Paediatric Ulcerative Colitis. Gut.

[35] Aziz B., Belaghi R., Huynh H., Jacobson K., Mack D.R., Deslandres C., Otley A., DeBruyn J., El-Matary W., Crowley E. (2025). Neutrophil-to-Lymphocyte Ratio at Diagnosis Predicts Colonoscopic Activity in Pediatric Inflammatory Bowel Diseases. Clin. Transl. Gastroenterol..

[36] Yoon J.Y., Cheon J.H., Park J.J., Hong S.P., Kim T.I., Kim W.H. (2011). Clinical Outcomes and Factors for Response Prediction after the First Course of Corticosteroid Therapy in Patients with Active Ulcerative Colitis. J. Gastroenterol. Hepatol..

[37] Torres J., Caprioli F., Katsanos K.H., Lobatón T., Micic D., Zerôncio M., Van Assche G., Lee J.C., Lindsay J.O., Rubin D.T. (2016). Predicting Outcomes to Optimize Disease Management in Inflammatory Bowel Diseases. J. Crohn’s Colitis.

[38] Song D.J., Shen J., Chen M.H., Liu Z.J., Cao Q., Hu P.J., Gao X., Qian J.M., Wu K.C., Lai L.J. (2021). Association of Serum Immunoglobulins Levels With Specific Disease Phenotypes of Crohn’s Disease: A Multicenter Analysis in China. Front. Med..

[39] López P., de Paz B., Rodríguez-Carrio J., Hevia A., Sánchez B., Margolles A., Suárez A. (2016). Th17 Responses and Natural IgM Antibodies Are Related to Gut Microbiota Composition in Systemic Lupus Erythematosus Patients. Sci. Rep..

[40] Grönwall C., Hardt U., Gustafsson J.T., Elvin K., Jensen-Urstad K., Kvarnström M., Grosso G., Rönnelid J., Padykov L., Gunnarsson I. (2017). Depressed Serum IgM Levels in SLE Are Restricted to Defined Subgroups. Clin. Immunol..

[41] Silverberg M.S., Mirea L., Bull S.B., Murphy J.E., Steinhart A.H., Greenberg G.R., McLeod R.S., Cohen Z., Wade J.A., Siminovitch K.A. (2003). A Population- and Family-Based Study of Canadian Families Reveals Association of HLA DRB1*0103 with Colonic Involvement in Inflammatory Bowel Disease. Inflamm. Bowel Dis..

[42] Preisker S., Brethack A.-K., Bokemeyer A., Bettenworth D., Sina C., Derer S. (2019). Crohn’s Disease Patients in Remission Display an Enhanced Intestinal IgM^+^ B Cell Count in Concert with a Strong Activation of the Intestinal Complement System. Cells.

